# Utilization of iron fillings solid waste for optimum biodiesel production

**DOI:** 10.3389/fchem.2024.1404107

**Published:** 2024-05-30

**Authors:** Fady I. El-Bayoumy, Ahmed I. Osman, David W. Rooney, Mai H. Roushdy

**Affiliations:** ^1^ Chemical Engineering Department, Faculty of Engineering, The British University in Egypt (BUE), El-Sherouk City, Egypt; ^2^ School of Chemistry and Chemical Engineering, Queen’s University Belfast, Belfast, United Kingdom

**Keywords:** iron filings, solid waste, transesterification, heterogeneous catalysis, biodiesel, waste valorization

## Abstract

This study explores the innovative application of iron filings solid waste, a byproduct from mechanical workshops, as a heterogeneous catalyst in the production of biodiesel from waste cooking oil. Focusing on sustainability and waste valorization, the research presents a dual-benefit approach: addressing the environmental issue of solid waste disposal while contributing to the renewable energy sector. Particle size distribution analysis, X-ray diffraction (XRD), scanning electron microscopy (SEM), X-ray fluorescence (XRF), Thermal analysis (TG-DTA), and FTIR analysis were used to characterize the iron filings. The response surface methodology (RSM) was used to guide a series of experiments that were conducted to identify the optimum transesterification settings. Important factors that greatly affect the production of biodiesel are identified by the study, including catalyst loading, reaction time, methanol-to-oil ratio, reaction temperature, and stirring rate. The catalyst proved to be successful as evidenced by the 96.4% biodiesel conversion efficiency attained under ideal conditions. The iron filings catalyst’s reusability was evaluated, demonstrating its potential for numerous applications without noticeably decreasing activity. This work offers a road towards more environmentally friendly and sustainable chemical processes in energy production by making a strong argument for using industrial solid waste as a catalyst in the biodiesel manufacturing process.

## Graphical Abstract

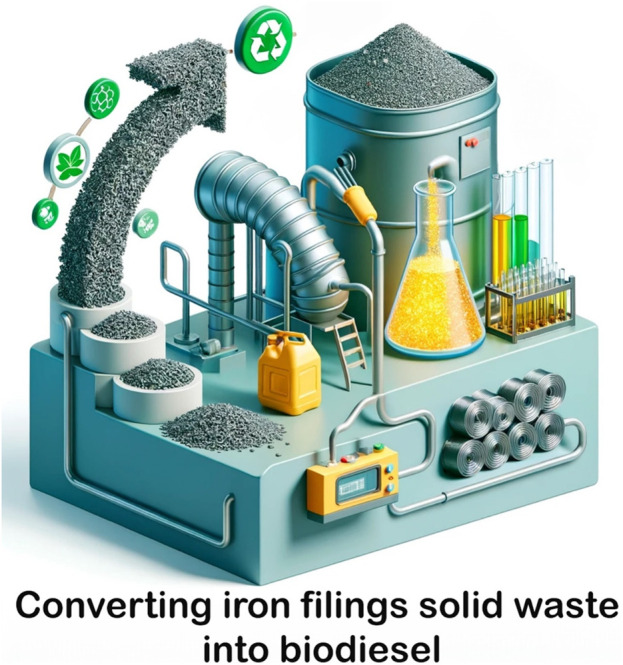

## 1 Introduction

In the context of modern environmental and energy challenges, the efficient management and valorization of solid waste emerge as critical areas of focus. Defined by the Resource Conservation and Recovery Act (RCRA), solid waste encompasses a broad spectrum of discarded materials from residential, commercial, industrial, and agricultural sources (Epa and O. of Resource Conservation, 2014) These materials range in composition from semi-solid to liquid and gaseous states, each with distinct properties such as corrosivity, ignitability, reactivity, and toxicity, which can pose significant environmental and health risks (Dehghani et al., 2021). The decomposition of garbage into its chemical components significantly contributes to environmental contamination, a prominent concern in developing nations where the reuse of landfills is limited due to financial constraints, resulting in unmet environmental health standards. Moreover, the emission of gases from decomposing waste poses a considerable environmental risk. In the anaerobic conditions of landfills, bacteria thrive, producing methane as a byproduct, further exacerbating the issue (Abdel-Shafy and Mansour, 2018).

Among the various types of industrial byproducts, iron filings—ferromagnetic fragments generated from the mechanical processing of steel and iron—represent a substantial component of solid waste. Traditionally viewed as environmental pollutants due to their disposal challenges, these materials are now being re-evaluated for their potential in sustainable applications, particularly in the realm of green chemistry and energy production (Jabar Hussain and Al-Khafaji, 2022).

The heavy reliance on large amounts of human-generated energy, mirroring the characteristics of natural gases and fuels, has led to a notable increase in the global price of petroleum oil. Such energy sources come with intrinsic limitations and are only viable for limited periods. This reality has spurred intensive research into alternative, renewable fuels. Moreover, the widespread use of petrochemical oils, natural gas, and coal plays a significant role in climate change and environmental pollution. This situation underscores the critical need to transition to more sustainable and cleaner energy sources (Edenhofer et al., 2012). The escalating global demand for energy, coupled with the environmental impact of conventional fossil fuels, underscores the urgency for alternative, sustainable energy sources. Biofuels, such as biodiesel derived from biomass feedstock or waste oils, offer a promising solution (Dahman et al., 2019; Zhang et al., 2024). Biodiesel production, specifically through the process of transesterification, transforms fats or oils into a cleaner fuel alternative, potentially achieving carbon neutrality by balancing CO_2_ emissions with absorption during biomass growth.

However, the biodiesel production process can be further optimized by leveraging solid wastes as catalysts in a heterogeneously catalyzed process. Heterogeneous catalysis offers distinct advantages over its homogeneous counterpart, including ease of separation, potential for catalyst reuse, and enhanced reaction efficiencies (Weldeslase et al., 2023; Ulukardesler, 2023; Veluru et al., 2022; Salihu et al., 2021; Nixon et al., 2022; Hasan and Ratnam, 2022; Shaban, 2012; Elgharbawy et al., 2021; Suzihaque et al., 2022; Monika and Pathak, 2023; A Alotaibi et al., 2024). Several researchers have investigated the use of solid waste as a catalyst in the synthesis of biodiesel. Eggshells, which are mostly calcium carbonate, have been discovered to be effective as catalysts in the biodiesel transesterification process. Waste chicken feathers have also been explored as potential acidic catalysts since they contain the protein keratin. Steel and dust solid wastes resulting from electric arc furnaces are also utilized as a perfect biodiesel catalyst. Waste mollusks and crabs’ shells, sanitary ware waste, ductile cast iron solid waste, kitchen food waste, banana peels, spent coffee grounds, geothermal solid waste, waste bull bone, Tomato pomace Waste, biocatalyst, and biomass-based SO_3_H-functionalized graphene are used as a biodiesel catalyst (Hijosa-Valsero et al., 2019; Widayat et al., 2023; Haile, 2014; Meka Kedir and Girma Asere, 2022; Barik et al., 2018; Roushdy, 2022a; El-Khashab et al., 2022; Roushdy, 2022b; El-Gendy et al., 2014; Ali et al., 2021; Khodary et al., 2023; Saravanan et al., 2024; Huang et al., 2023a; Carvalho de Melo et al., 2023; Huang et al., 2023b). Exploring the use of iron filings to be used a heterogenous biodiesel catalyst aligns with the principles of circular economy and green chemistry, offering an innovative approach to waste valorization and sustainable energy production.

This work explores the use of solid waste iron filings as a heterogeneous catalyst for the generation of biodiesel from waste cooking oil. The goal of this research seeks to improve the production of renewable biofuels and contribute to the sustainable management of solid waste by examining important reaction parameters and using response surface methods for process optimization. The goal of the study is to demonstrate the double advantages of this strategy are reducing waste disposal problems and supporting renewable energy sources by looking at them through the lenses of environmental preservation and energy sustainability.

## 2 Materials and methods

### 2.1 The research raw materials

The following are the raw substances used in this study:

(a) Iron filings solid waste which is was collected from a steel industry located in Egypt.
(b) Methanol (MeOH) with a concentration of 99% was acquired from ALFA Chemical Group.
(c) Waste sunflower cooking oil acquired from an Egyptian restaurant.

### 2.2 Characterization of solid waste

The solid waste was characterized using the same method and standards that were mentioned in the previous research paper (El-Khashab et al., 2022) in addition to Scanning Electron Microscopy (SEM), Thermogravimetry-differential thermal analysis (TG-DTA), and Fourier transform infrared spectrophotometer (FTIR) with details mentioned in Table 1.

**TABLE 1:** Characterization methods for the utilized solid waste.

Method	Objective	Test description	Used standard
Scanning Electron Microscopy (SEM)	Used to examine the microstructure and morphology/texture of a material	It involves the creation of a variety of signals at the solid object’s surface by the utilization of a focused beam that contains electrons at high energy levels. These signals provide data about the sample, such as its crystalline structure, chemical composition, and alignment of the components present in the sample	ISO/TS 21383:2021 (Microbeam analysis, 2021)
Thermogravimetry-differential thermal analysis (TG-DTA)	Used to determine the thermal stability of the solid waste which is mainly α-Fe_2_O_3_	It was assessed using the PerkinElmer TGA 4000 (Netherlands). For the analysis, nitrogen gas was employed at operating parameters of 60 mL/min flow rate and 10°C/min heating rate from 25 to 1,000°C	Cheremisinoff (1996)
Fourier transform infrared spectrophotometer (FTIR)	The surface functional groups of the used solid waste are determined using Fourier-transform infrared (FTIR) analysis (Vertex 70 RAM II, Germany)	Potassium bromide was used to pelletize the samples, and spectra were obtained by accumulating 32 total scans at a resolution of 4 cm^−1^ in the wavenumber range of 450–4,000 cm^−1^	Undavalli et al. (2021)

### 2.3 Arrangement and assembly of SFWCO

Sunflower waste cooking oil (SFWCO), once deemed worthless by many households, can be effectively purified to eliminate any suspended particulates, fried food remnants, and other contaminants. The purification process involves utilizing a centrifuge and filter to separate the impurities from the oil. Following this step, SFWCO undergoes 2 h of drying at 105°C to extract any remaining water content. This treatment paves the way for its efficient reuse in various applications.

### 2.4 Biodiesel production

**FIGURE 1:**
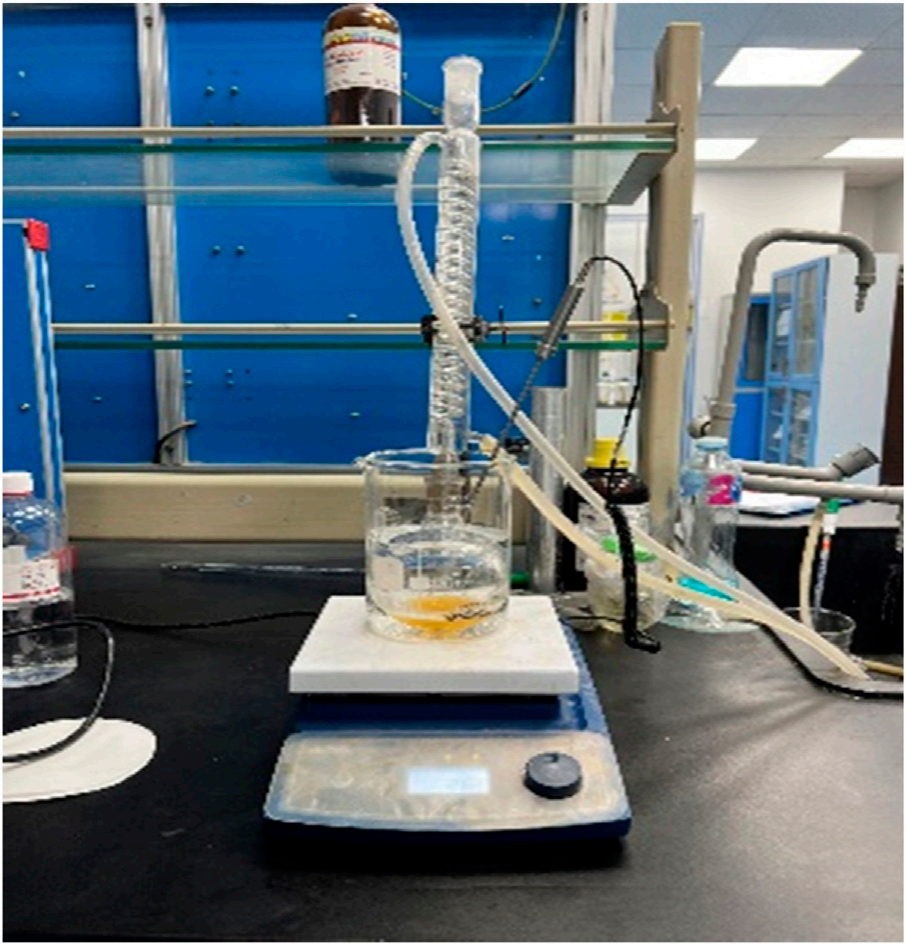
Experimental setup.

The trial step that was utilized for the creation of biodiesel, as shown in Figure 1, can be illustrated as follows: The trans-esterification technique of biodiesel synthesis was used, and the reaction happened in a 250 mL round bottom flask coupled with a reflux condenser to prevent methanol escape. The flask was placed in a 1,000 mL beaker filled with water to serve as a heating medium, along with a thermometer placed in the water for measuring temperature while accounting for an additional 5°C higher than that of the flask.

To create biodiesel, the necessary components such as methanol, catalyst (iron filings), and oil were efficiently added to a batch reactor. Proper attention was paid to maintaining the required catalyst concentration and adhering to the right m:o ratio. Following this, the temperature of the reaction was appropriately adjusted while starting and setting the timer for a specific duration. Once complete, a filter media was used to remove the solid catalyst, which then left glycerol behind. With a separating funnel, any methanol that was found in excess amounts was completely removed through an 80°C—30-min drying process until finally, we could calculate how much biodiesel had been made based on its weight ratio when compared against SFWCO that was initially used. The conversion was calculated using Eq. 1 .

$$Biodiesel\;Conversion\%=\frac{Weight\;of\;biodiesel\;produced}{Weight\;of\;sunflower\;oil}x100$$ (1)

### 2.5 Experimental design

To make a perfect design for the experimental work, the surface methodological technique (RSM) was used, along with the aid of Design Expert software version 13 for detailed analysis (Montgomery, 2013). RSM includes an array of statistical and mathematical approaches for developing empirical models. RSM aims to relate a response to the amounts of various input factors/variables that impact it through suitable experiment analysis and design. The reaction variables are labelled as A, B, C, D, and E, as indicated in Table 2. Biodiesel and glycerol conversions have been selected as reaction responses. The choice of reaction conditions and ranges were also chosen based on previous research (El-Khashab et al., 2022; Khodary et al., 2023; El-Sheltawy and Al-Sakkari, 2016; Talha and Sulaiman, 2016; Ling et al., 2019; Refaat, 2011).

**TABLE 2:** Biodiesel reaction variables.

Reaction variable	Symbol	Ranges
Reaction time (hr)	A	1–4
Methanol: Oil (Molar Ratio)	B	5–20
Catalyst loading (wt.%)	C	1–5
Temperature (^o^C)	D	50–70
Stirring Rate (RPM)	E	200–800

A total of forty-three trials had been built in the matrix of uncertainty as a means of reducing the number of experiments necessary. The experimental runs were set up randomly, and the responses were calculated based on the outcomes of each trial. The design procedures of the experiment were identified and employed by the utilization of DoE.

In the production of biodiesel, the M:O molar ratio is crucial. To convert one mole of TG into FAME or biodiesel, three moles of methanol are needed. This requires additional methanol to maintain the reaction rate. A molar ratio of M:O greater than 3:1 is necessary, with a range of 5:1 to 20:1 to analyze the impact of excess methanol on biodiesel yield. The yield of biodiesel directly increases with temperature between 50 and 70°C. This could be explained by the oil’s decreased viscosity. However, this effect becomes insignificant beyond 70°C.

Optimal conditions for biodiesel production are reaction time of 4 h, methanol to oil ratio of 20, catalyst loading of 5%, stirring rate of 800 rpm, and temperature of 70°C, resulting in a 99.58% yield. Lower limit conditions are reaction time of 1 h, methanol-to-oil ratio of 5, catalyst loading of 1%, stirring rate of 200 rpm, and temperature of 50°C, resulting in an 82.9% yield. Any increase in optimal conditions or decrease in lower limit conditions will not lead to significant yield improvement.

### 2.6 Optimum biodiesel sample analysis

To ascertain that the resulting product is biodiesel, the following tests were done using ASTM standards methods as mentioned in Table 3 then the results were compared with the biodiesel American and British standards.

**TABLE 3:** Optimum biodiesel sample analysis.

Tests	Description		Compared standards
Biodiesel composition determination using GC or gas chromatography	Determine the quantity of total fatty acid methyl ester (FAME), glycerol, and triglycerides in a biodiesel sample		EN 14103 (Biodiesel, 2011) EN 14105 (Biodiesel and Glycerol, 2011)
Physicochemical properties determination	Determine the following properties:	The used standards:	EN 14214 (Liquid Petroleum Products, 2013) ASTM D6751 (A. D6751-15c, 2010)
	1- Kinematic Viscosity at 40°C	ASTM D445 (ASTM D445-06, 2008)	
	2- Density at 15°C (g/cm^3^)	ASTM D4052 (D40052-15, 2013)	
	3- Calorific Value (mJ/kg)	ASTM D5865 (Mahapatra, 2016)	
	4- Pour Point (°C)	ASTM D97 (Edition, 2013)	
	5- Cloud Point (°C)	ASTM D97 (Edition, 2013)	
	6- Flash point (°C)	ASTM D93 (Products, 2004)	

### 2.7 Reusability test of iron filings solid waste catalyst

Under the optimal conditions, a reusability test was performed. The reaction product was filtered after the reaction was completed to eliminate the catalyst. Table 4 summarizes the approach employed. The catalyst strength and efficiency were determined by calculating the reaction conversion at the stage of reutilization.

**TABLE 4:** Reusability test.

Step	Details
1. Removal of contaminants via washing chemical treatment method	The washing was done by the utilization of methanol
2. Drying	The catalyst was dried at a temperature of 80°C for a duration of 30 min

## 3 Results and discussion

### 3.1 Iron filings characterization

#### 3.1.1 X-ray fluorescence for chemical analysis

Table 5 shows the chemical assessment of the iron-filled solid waste. The results show that the waste is largely made of 96.15% Fe_2_O_3_, with only trace levels of other oxides, as expected. CO_2_ generation, the loss of combustible organic products, and moisture content loss might account for the loss observed during igniting. These findings provide persuasive proof that solid waste that contains iron has enormous potential as a biodiesel catalyst. Prior research has also shown that Fe_2_O_3_ is effective as a component for biodiesel, which supports its usage in this study.

**TABLE 5:** Chemical analytics of iron filings.

Oxide	Percentage, %
Fe_2_O_3_	96.15
SiO_2_	1.25
CaO	0.91
MnO	0.49
TiO_2_	0.07
Na_2_O	0.04
P_2_O_5_	0.04
MgO	0.01
Al_2_O_3_	<0.01
K_2_O	<0.01
Cl	<0.01
SO_3_	<0.01
Loss on Ignition	0.7

#### 3.1.2 Mineralogical examination

**FIGURE 2:**
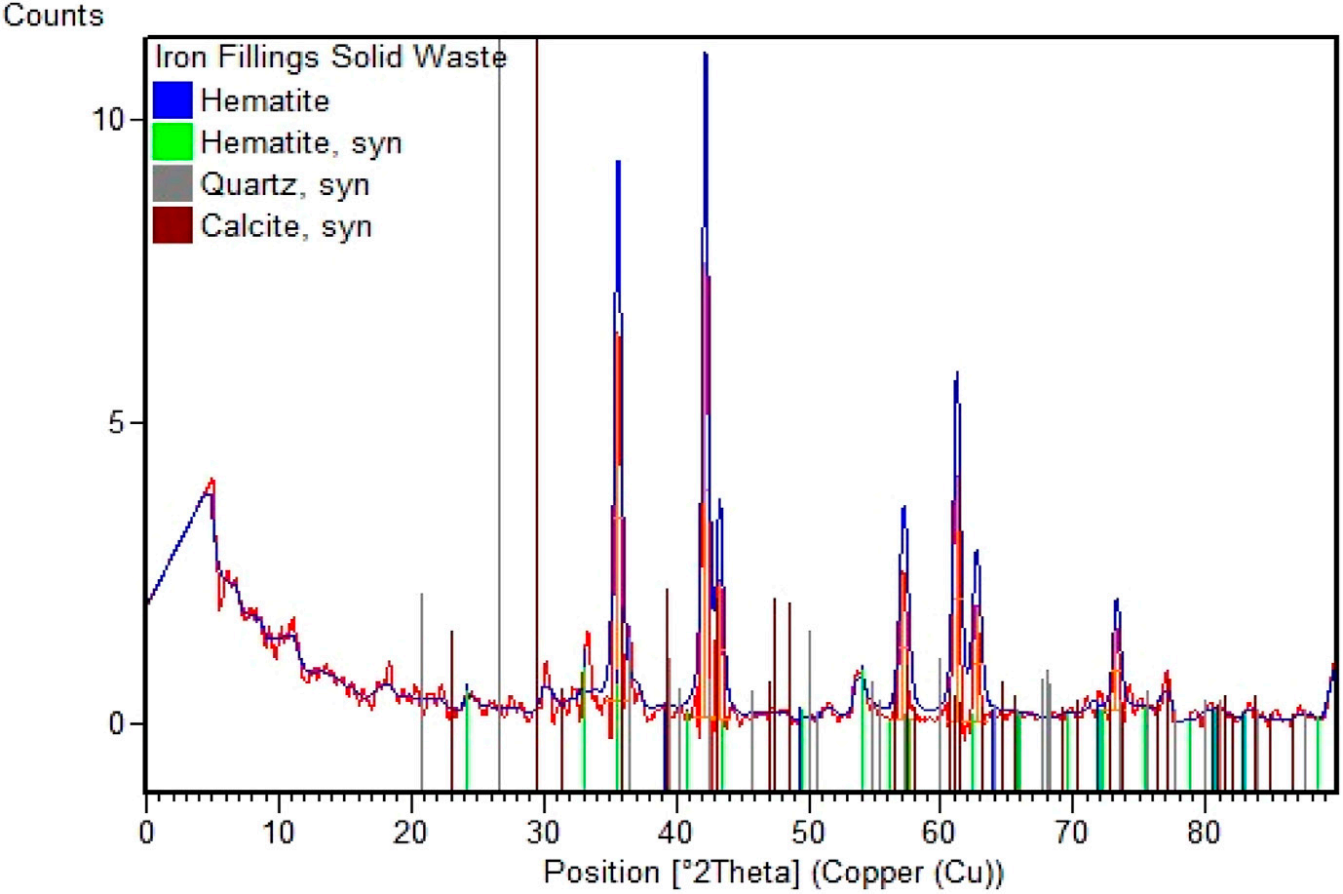
Mineralogical examination of the iron filings.

The conclusions of the iron fillings’ mineralogy assessment are shown in Figure 2. According to the results of the investigation, the predominant phase present is Periclase, namely, hematite (α-Fe_2_O_3_), which has a rhombohedral structure. The blue crystal is known as “JCPDS-00-024-0072,” while the green crystal is known as “JCPDS-00-033-0664,” also known as burnt ochre Hematite. The unusual form of these hematite crystals suggests that solid waste has significant promise as a biodiesel catalyst. Minor phases of quartz and calcite were also discovered throughout the study. These X-ray diffraction (XRD) study findings confirm and complement the X-ray fluorescence (XRF) analysis results, further enhancing solid waste characterization.

#### 3.1.3 Screen analysis

**FIGURE 3:**
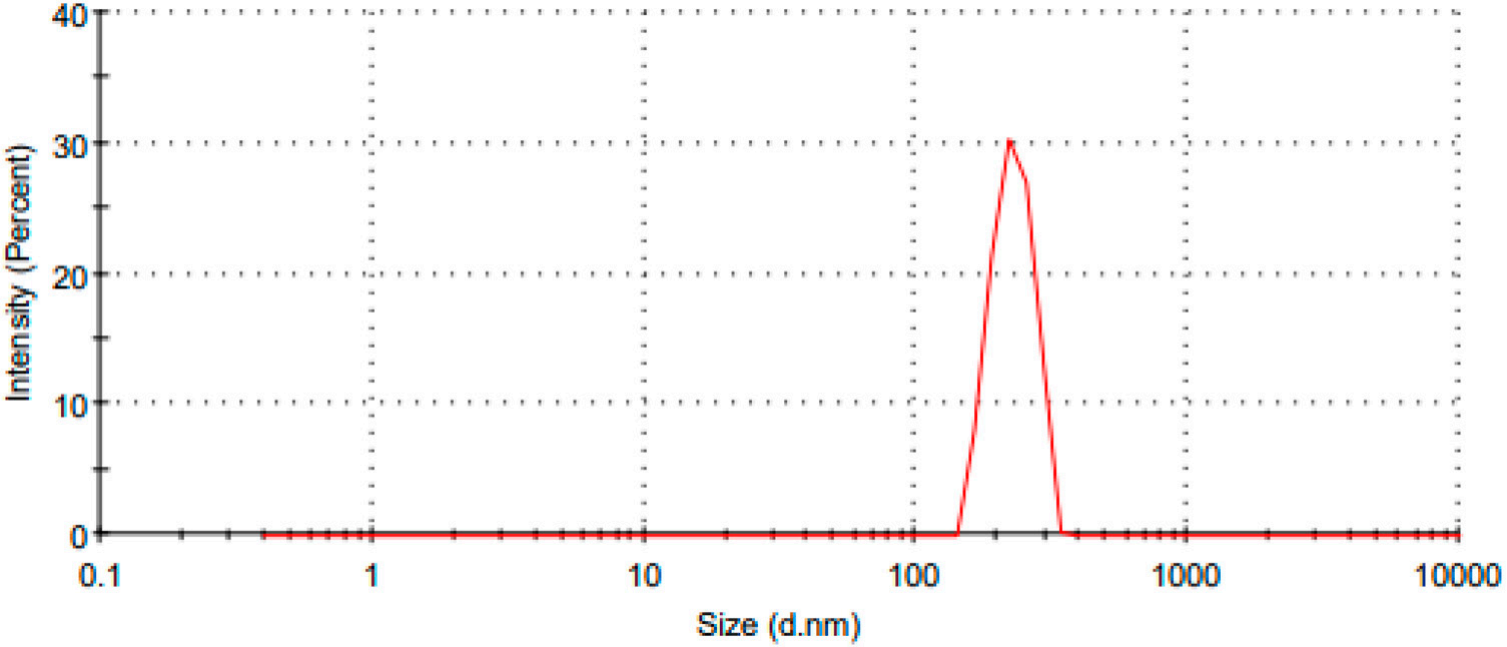
Cumulative screen examination curve of the iron filings.

The Malvern device was used to determine the size of the catalyst particles, which resulted in an average size of 549.9 nm, as shown in Figure 3. This observation indicates that the catalyst has a large surface area, which is critical for its effectiveness in promoting the reaction. A narrow particle size distribution and small particle size suggest a more reactive surface area available. As a result of its large surface area, this catalyst is expected to exhibit extraordinary activity, making it an excellent choice for the planned application.

#### 3.1.4 Microstructure and morphological analysis

**FIGURE 4:**
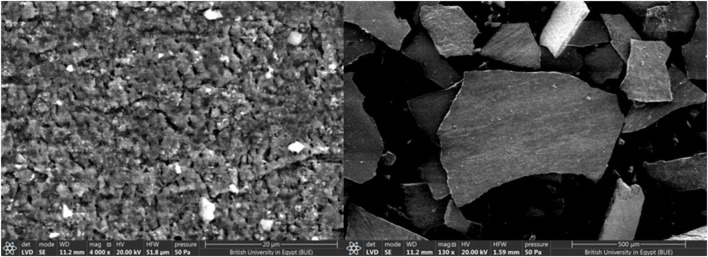
SEM examination for iron fillings.

Figure 4 illustrates that the catalyst’s surface is non-uniform, with varied shapes and active centers, indicating that it would be an excellent biodiesel catalyst.

#### 3.1.5 Thermogravimetry-differential thermal analysis (TG-DTA)

**FIGURE 5:**
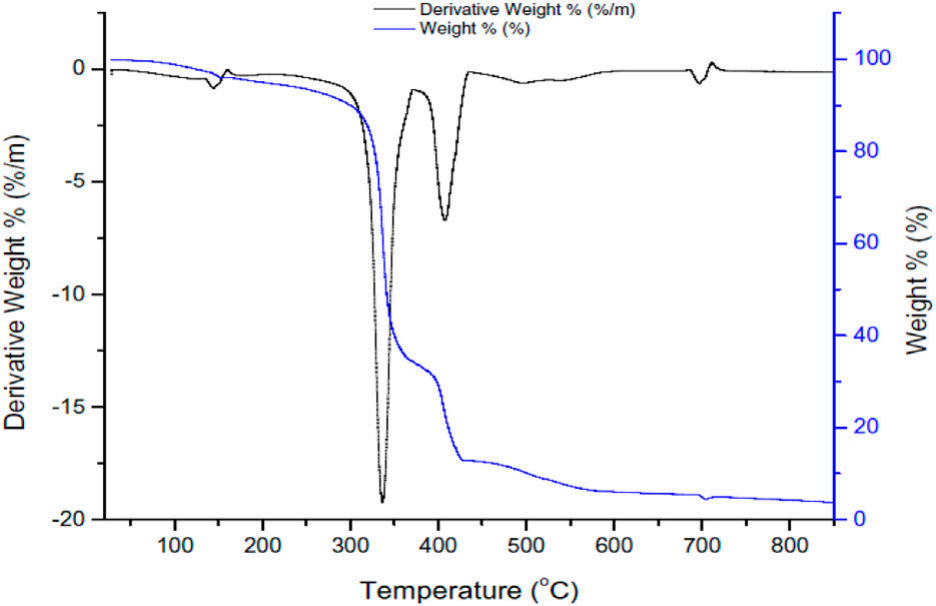
Thermal analysis (TG-TDA) of the iron fillings.

Figure 5 shows the TG-DTA analyses for the solid waste.

• There are three main regions of decomposition shown in the thermograph as follows and after these losses of weight then above 600°C, the sample remains stable.
  1- A weight loss of 10 wt% at 300°C because of weakly bonded or physically adsorbed water removal (Darezereshki, 2011).
  2- A weight loss of 55 wt% at 320°C–370°C and another 25 wt% weight loss at 370°C–420°C because of chemically adsorbed water removal because of the silanol group (Si–OH) condensation (Darezereshki, 2011; Qu et al., 2017).
  3- A weight loss of 10 wt% at 420°C–600°C because of chemical components decomposition and crystalline phase transformation (Darezereshki, 2011).
• The DTA graph shows endothermic peaks as follows and after these peaks, the sample is stable as the curve becomes parallel to the x-axis.
  1- A peak at 150°C corresponds to 0.5 wt% weight loss due to the loss of moisture from the solid waste sample.
  2- A peak at 300°C–370°C corresponds to 18 wt% weight loss because of the loss of combustible organic products.
  3- A peak at 370°C–440°C corresponds to 5 wt% weight loss because of the transition phase.
  4- A peak at 680°C.

It is noticed because of the crystallization and phase transition there is no associate signal was noticed in TGA when compared with the DTA curve. The obtained results were comparable with the results obtained by the thermal analysis of α-Fe_2_O_3_ as mentioned by Lassoued et al. (2017a) and Waseem et al. (2014).

#### 3.1.6 Fourier transform infrared spectrophotometer (FTIR)

**FIGURE 6:**
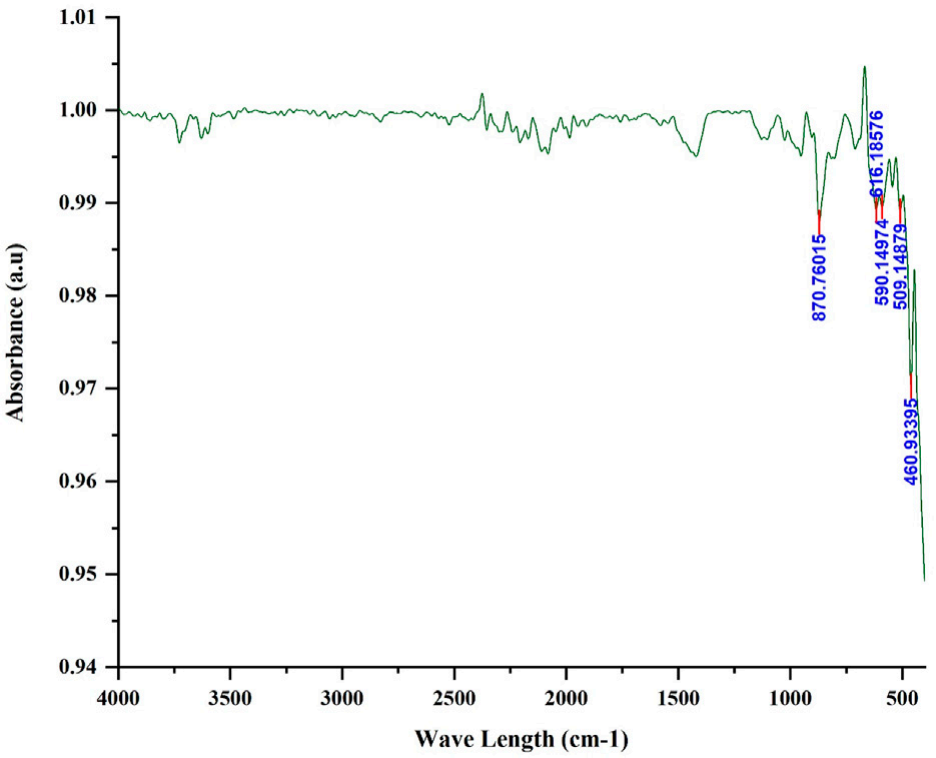
FTIR of solid waste.

Figure 6 shows the FTIR for the iron filling within the range of 400–4,000 cm^−1^. The list of peaks is shown in the following table. Based on FTIR analysis the strong band below 700 cm^−1^ is assigned for Fe-O stretching mode (Farahmandjou and Soflaee, 2015). The absorption peaks at the wavelengths of 460.9, 509.1, and 616.2 cm^−1^ correspond to the Fe-O stretching mode of Fe_2_O_3_ and the vibration of Fe-O in the rhombohedral lattice of hematite which is an important characteristic of the crystalline of α-Fe_2_O_3_ compound (Lassoued et al., 2017b; Jing and Wu, 2004). The absorption peak at the wavelength of 870.8 may be due to the presence of an oxygen-containing function group of calcite (Gawad et al., 2022) or aromatic C-H stretching vibration because of the presence of organic impurities in the sample (Mansour et al., 2022).

### 3.2 Process modelling using design expert programme

The previous experiments allowed us to calculate the rates of conversion of both biodiesel & glycerol. With the utilization of Design Expert, models were created by showing how process or reaction factors impact biodiesel and glycerol conversion. The ANOVA approach was utilized with a 95% confidence level to assess the significance and appropriateness of these models, taking P and F values into account. The findings of the investigation indicated that the quadratic model showed the highest efficiency in predicting biodiesel conversion and the most favorable outcomes for glycerol conversion. Nevertheless, several components within the models were considered statistically insignificant as their *p*-values exceeded the threshold of 0.05.

**FIGURE 7:**
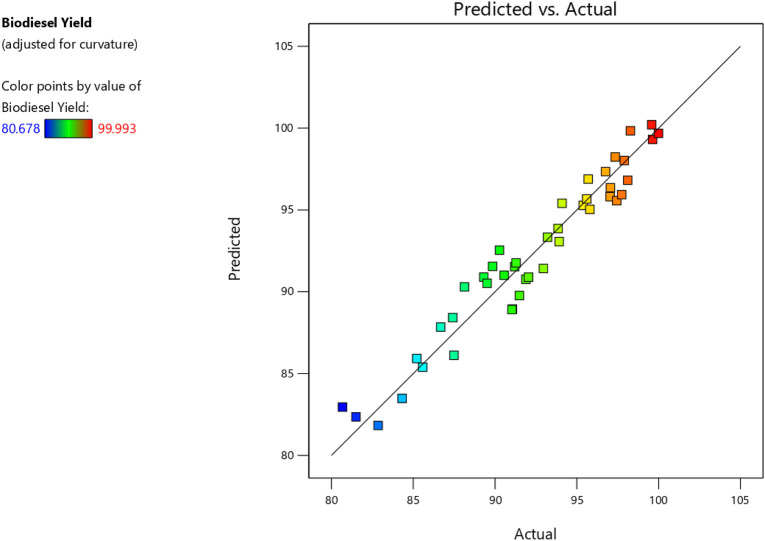
Actual and predicted biodiesel yield.

**FIGURE 8:**
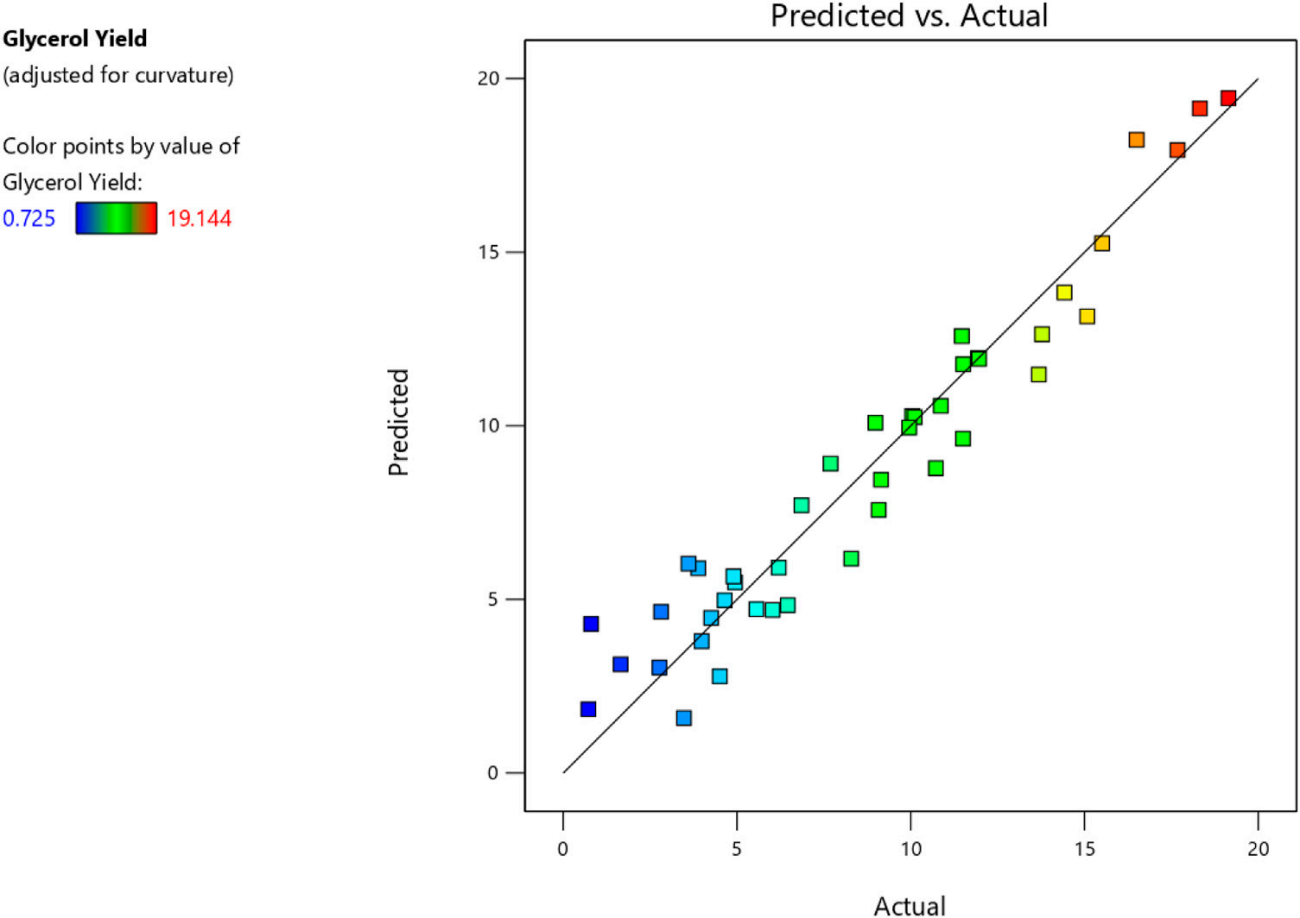
Actual and predicted glycerol yield.

Consequently, these unnecessary components were eliminated, resulting in simplified versions of the models expressed by Eqs 2, 3. Furthermore, the ANOVA analysis is summarized in Tables 6, 7. Finally, Figures 7, 8 compare estimated and experimental data for conversions of biodiesel and glycerol. The reasonable consistency seen in both figures and R values in Tables 6, 7 verifies the models’ appropriateness.

$$X=-0.916\;A+0.611\;B+0.132\;C+0.164\;D+0.009\;E+0.032\;AD-0.0015\;AE-0.0004\;BE-0.00009\;DE+{0.00002\;E}^{2}+68.85$$ (2)

$$Y=+0.177\;A-0.617\;B-0.3\;C-0.319\;D+0.017\;E-0.006\;AD+0.0002\;AE+0.0005\;BE+0.0002\;DE-0.00002{\;E}^{2}+40.108$$ (3)

**TABLE 6:** Analysis of variance analysis results for biodiesel response (Reduced Quadratic Model).

Item	F-value	*p*-value
Model	50.72	<0.0001
A-Reaction Time	1.25	0.2717
B-Methanol: Oil Ratio	152.91	<0.0001
C-Catalyst Loading	1.30	0.2632
D-Reaction Temperature	66.59	<0.0001
E-Stirring Rate	62.92	<0.0001
AD	4.55	0.0485
AE	6.93	0.0129
BE	13.20	0.0010
DE	5.12	0.02981
E^2^	8.62	0.0061
Model Accuracy	*R* ^2^	0.9407
	Predicted *R* ^2^	0.8919
	Adjusted *R* ^2^	0.9221

**TABLE 7:** Analysis of variance analysis results for glycerol response (Reduced Quadratic Model).

Item	F-value	*p*-value
Model	40.66	<0.0001
A-Reaction Time	1.2242	0.6391
B-Methanol: Oil Ratio	93.44	<0.0001
C-Catalyst Loading	5.82	0.0218
D-Reaction Temperature	52.65	<0.0001
E-Stirring Rate	53.14	<0.0001
AD	5.1276	0.04232
AE	5.1572	0.04944
BE	19.44	0.0001
DE	6.37	0.0168
E^2^	5.89	0.0210
Model Accuracy	*R* ^2^	0.9270
	Predicted *R* ^2^	0.8619
	Adjusted *R* ^2^	0.9042

The variable X represents biodiesel conversion in the context of the study, whereas the variable Y represents glycerol conversion. Both conversions are influenced by the reaction variables. All these reaction parameters were shown to have a positive stimulus on the conversion of biodiesel, indicating that increasing these components improves the conversion process. They have an adverse influence on the conversion of glycerol, implying that greater values of these parameters result in lower conversion of glycerol. In the following Table 8, Actual and predicted results for Biodiesel and glycerol yield with the experimental errors according to the suggested models. As shown in Table 8 the predicted values and the experimental or actual values of the responses are near to each other with a small absolute error rate for each run and a small mean error of 1.14 and 4.48 for biodiesel and glycerol yield responses respectively.

**TABLE 8:** Actual and predicted results for biodiesel and glycerol yield with the errors.

Run order	Reaction parameters					Biodiesel yield				Glycerol yield			
	A	B	C	D	E	Actual value	Predicted value	Residual	% error	Actual value	Predicted value	Residual	% error
1	1	5	1	50	200	82.86	81.83	1.03	1.24	19.14	19.44	−0.3	1.57
2	4	5	1	50	200	80.68	82.95	−2.27	2.81	18.32	19.14	−0.82	4.48
3	1	20	1	50	200	91.49	89.77	1.72	1.88	11.51	11.77	−0.26	2.26
4	4	20	1	50	200	89.31	90.89	−1.58	1.77	13.69	13.48	0.21	1.53
5	1	5	5	50	200	81.5	82.36	−0.86	1.06	16.5	17.24	−0.74	4.48
6	4	5	5	50	200	84.32	83.48	0.84	1.00	17.68	17.94	−0.26	1.47
7	1	20	5	50	200	88.14	90.3	−2.16	2.45	10.87	10.57	0.3	2.76
8	4	20	5	50	200	92.96	91.42	1.54	1.66	10.04	10.28	−0.24	2.39
9	1	5	1	70	200	85.58	85.39	0.19	0.22	14.42	13.84	0.58	4.02
10	4	5	1	70	200	87.42	88.42	−1	1.14	15.08	14.15	0.93	6.17
11	1	20	1	70	200	93.21	93.33	−0.12	0.13	8.29	8.17	0.12	1.45
12	4	20	1	70	200	97.05	96.36	0.69	0.71	4.95	5.29	−0.34	6.87
13	1	5	5	70	200	85.22	85.92	−0.7	0.82	13.78	12.64	1.14	8.27
14	4	5	5	70	200	91.06	88.95	2.11	2.32	11.94	11.95	−0.01	0.08
15	1	20	5	70	200	93.86	93.86	0	0.00	4.64	4.97	−0.33	7.11
16	4	20	5	70	200	95.7	96.89	−1.19	1.24	0.803	0.79	0.013	1.62
17	1	5	1	50	800	90.28	92.54	−2.26	2.50	10.72	9.78	0.94	8.77
18	4	5	1	50	800	90.56	91.01	−0.45	0.50	7.69	7.91	−0.22	2.86
19	1	20	1	50	800	98.11	96.82	1.29	1.31	3.89	3.9	−0.01	0.26
20	4	20	1	50	800	95.39	95.28	0.11	0.12	3.61	3.25	0.36	9.97
21	1	5	5	50	800	93.92	93.07	0.85	0.91	9.08	8.58	0.5	5.51
22	4	5	5	50	800	91.2	91.54	−0.34	0.37	6.86	7.51	−0.65	9.48
23	1	20	5	50	800	96.75	97.34	−0.59	0.61	6.03	5.7	0.33	5.47
24	4	20	5	50	800	97.04	95.81	1.23	1.27	6.46	5.83	0.63	9.75
25	1	5	1	70	800	95.8	95.04	0.76	0.79	6.2	5.92	0.28	4.52
26	4	5	1	70	800	94.1	95.41	−1.31	1.39	4.9	4.66	0.24	4.90
27	1	20	1	70	800	99.63	99.31	0.32	0.32	2.77	3.04	−0.27	9.75
28	4	20	1	70	800	99.99	99.68	0.31	0.31	4.51	4.78	−0.27	5.99
29	1	5	5	70	800	97.44	95.56	1.88	1.93	5.56	5.72	−0.16	2.88
30	4	5	5	70	800	97.74	95.93	1.81	1.85	4.26	4.46	−0.2	4.69
31	1	20	5	70	800	98.28	99.84	−1.56	1.59	0.725	0.74	−0.015	2.07
32	4	20	5	70	800	99.58	99.21	0.37	0.37	3.47	3.58	−0.11	3.17
33	1	12.5	3	60	500	89.52	90.52	−1	1.12	8.98	9.09	−0.11	1.22
34	6	12.5	3	60	500	91.29	91.76	−0.47	0.51	11.5	10.63	0.87	7.57
35	2.5	5	3	60	500	86.68	87.84	−1.16	1.34	11.47	12.58	−1.11	9.68
36	2.5	30	3	60	500	97.9	98.02	−0.12	0.12	3.99	3.8	0.19	4.76
37	2.5	12.5	2	60	500	91.89	90.76	1.13	1.23	10.11	10.25	−0.14	1.38
38	2.5	12.5	8	60	500	89.85	91.55	−1.7	1.89	9.15	8.45	0.7	7.65
39	2.5	12.5	3	36	500	87.49	86.12	1.37	1.57	15.51	15.26	0.25	1.61
40	2.5	12.5	3	84	500	95.6	95.67	−0.07	0.07	2.82	2.64	0.18	6.38
41	2.5	12.5	3	60	214	91.04	88.91	2.13	2.34	11.96	11.93	0.03	0.25
42	2.5	12.5	3	60	900	97.35	98.23	−0.88	0.90	1.65	1.53	0.12	7.27
43	2.5	12.5	3	60	500	92.05	90.89	1.16	1.26	9.95	9.95	0	0.00
						Average or mean error			1.14	Average or mean error			4.48

### 3.3 Relation between one reaction variable and both biodiesel and glycerol responses

**FIGURE 9:**
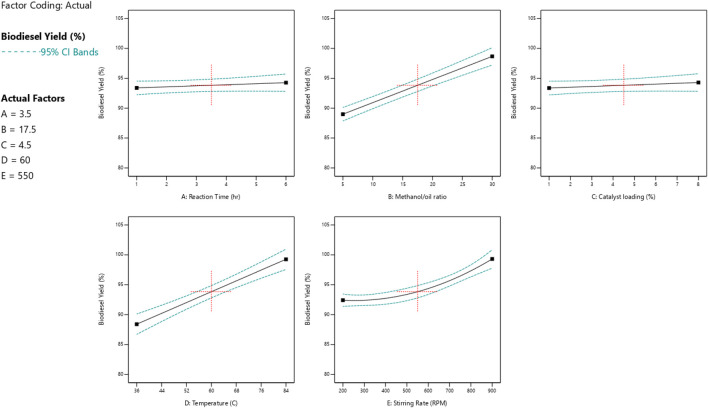
Reaction parameters influence the yield of biodiesel.

**FIGURE 10:**
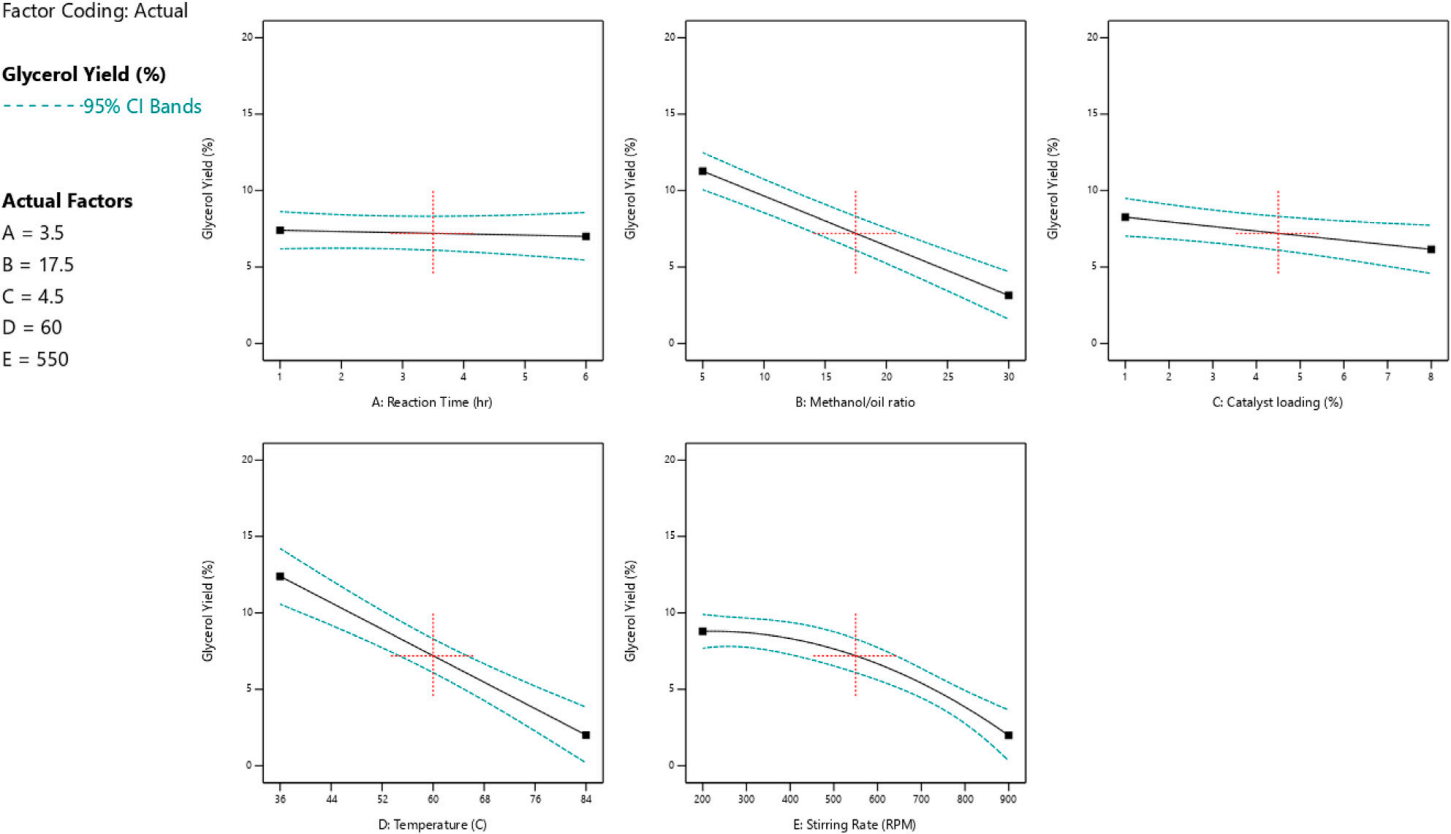
Reaction parameters influence the yield of glycerol.

The impact of each reaction parameter on the conversions of glycerol and biodiesel is displayed in Figures 9, 10. The amount of catalyst added to the reaction mixture and the reaction time has approximately no effect because it is not a significant factor, as indicated by the ANOVA analysis as they are non-significant factors. The two figures show that the reaction temperature, the M:O ratio, and the stirring rate have the greatest impact on both biodiesel and glycerol conversions.

### 3.4 Relation between all reaction variables and both biodiesel and glycerol responses

**FIGURE 11:**
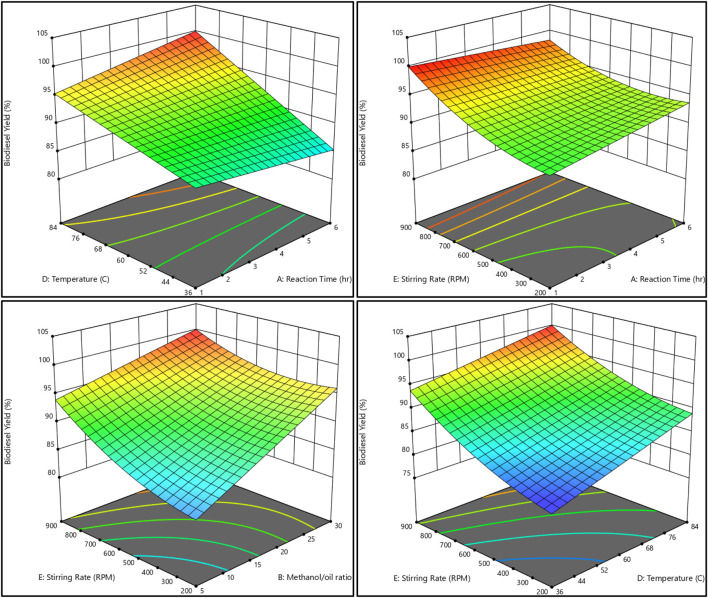
As a 3D surface graph, the relationship between the biodiesel yield, reaction temperature, reaction time, methanol to oil ratio, and stirring rate.

**FIGURE 12:**
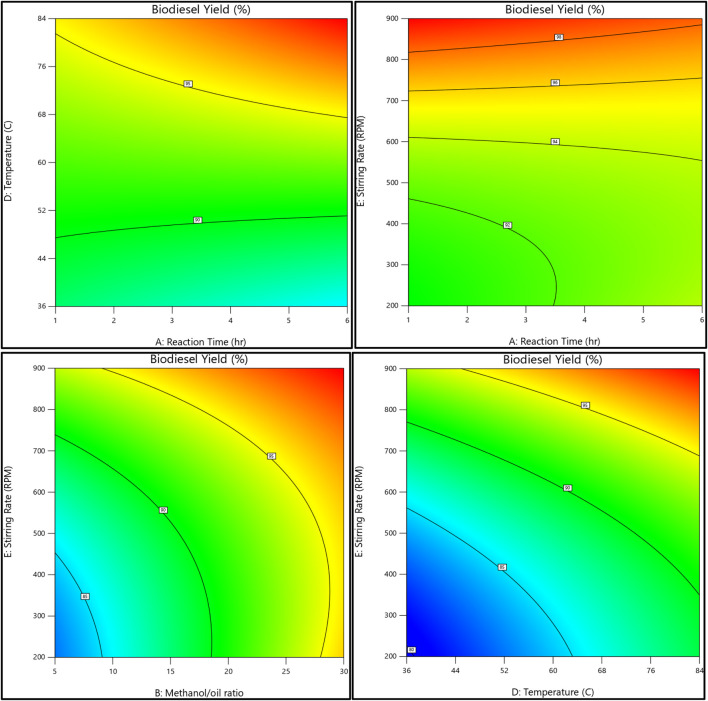
As a contour plot, the relationship between the biodiesel yield, reaction temperature, reaction time, methanol to oil ratio, and stirring rate.

**FIGURE 13:**
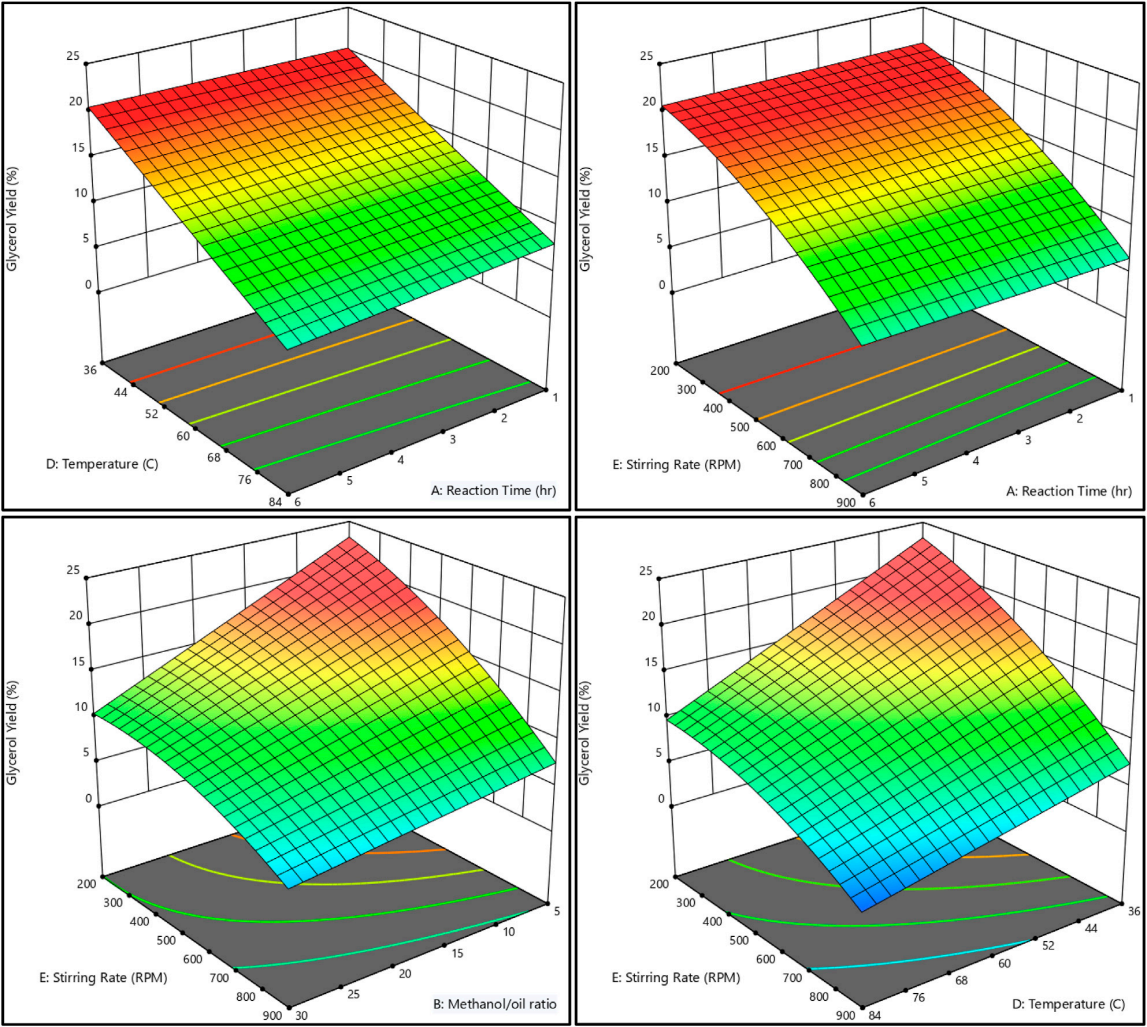
As a 3D surface graph, the relationship between the glycerol yield, reaction temperature, reaction time, methanol to oil ratio, and stirring rate.

**FIGURE 14:**
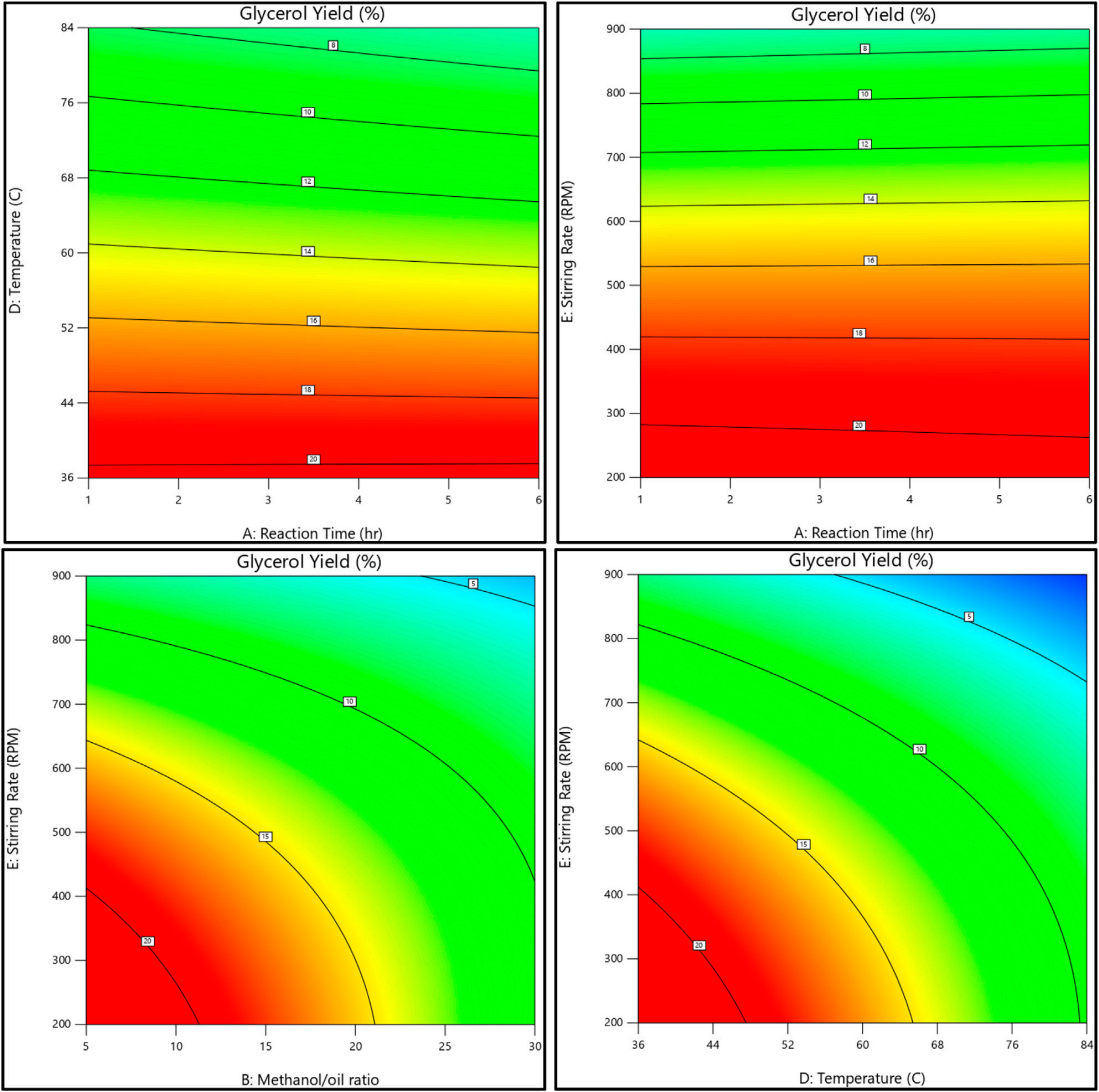
As a contour plot, the relationship between the glycerol yield, reaction temperature, reaction time, methanol to oil ratio, and stirring rate.

The relationship between the biodiesel yield and methanol, as well as the methanol-to-oil ratio, reaction temperature, reaction time, and stirring rate, is shown in Figures 11, 12. On the other hand, Figures 13, 14 illustrate the relationship between glycerol production and the methanol-to-oil ratio, reaction temperature, reaction time, and stirring rate.

### 3.5 Process optimization

The transesterification reaction optimization procedure sought to establish the best values for the independent variables, which included the M:O molar ratio, catalyst loading, reaction temperature, stirring rate, and reaction duration. These factors had an immediate impact on the dependent response variables, which were glycerol and biodiesel conversion. Design of experiment (Design Expert software) was utilized to make the optimization process. The program looked for the best settings to accomplish the intended response goals. A set of objectives was developed inside the program to discover the ideal circumstances for the independent variables. These goals were set with environmental and economic feasibility in mind. Temperature, stirring rate, and reaction time were tuned to be minimized to utilize the least amount of energy. This strategy is intended to enhance energy efficiency and minimize resource usage. The M:O molar ratio and the amount of catalyst, on the other hand, were controlled within a certain range, so any extra methanol might be collected for future use. Finally, the biodiesel output was set to be maximized, while the glycerol yield was to be minimized within the stated constraints of the independent variables’ objectives. Based on the previously stated goals, the design expert program developed 100 recommended solutions with varying degrees of desirability and then chose the best solution with the highest degree of desire. The best reaction conditions that lead to 96.4% biodiesel yield and 3.95% glycerol yield are reaction time equals 1 h, Methanol to oil ratio equals 30, catalyst loading 8%, temperature equals 52°C, and stirring rate equals 200 RPM.

### 3.6 Optimum biodiesel sample analysis

The physicochemical properties of this ideal sample were obtained through comparison to its guidelines ASTM D 6751 (A. D6751-15c, 2010) and EN14214 (Liquid Petroleum Products, 2013), as shown in Table 9. All measured qualities meet or exceed the relevant criteria. Table 10 shows the results of the best sample’s GC tests, as well as adherence to the guidelines of biodiesel EN 14105 (Biodiesel and Glycerol, 2011) and EN 14103 (Biodiesel, 2011).

**TABLE 9:** Physicochemical guidelines and properties of biodiesel.

Physicochemical	Method (standard)	Results	ASTM D67571	EN 14214
Kinematic Viscosity at 40°C	ASTM D445 (ASTM D445-06, 2008)	4.8	1.9–6	3.5-5.0
Calorific Value (mJ/kg)	ASTM D5865 (Mahapatra, 2016)	42.18		>32.9
Pour Point (°C)	ASTM D97 (Edition, 2013)	−20		
Flash point (°C)	ASTM D93 (Products, 2004)	150	>130	>101
Density at 15°C (g/cm^3^)	ASTM D4052 (D40052-15, 2013)	0.86		0.86-0.9
Cloud Point (°C)	ASTM D-97 (Edition, 2013)	−9		<-4

**TABLE 10:** Results and standards for GC analysis.

Composition	Specification	Results (%)
Total Fatty Acid Methyl Esters	more than 96.5%	96.70
Free Glycerol	less than 0.02	0.02
Total Glycerol	less than 0.25	0.02
Mono-Glyceride	less than 0.08	0.02
Di-Glyceride	less than 0.02	0.01
Tri-Glyceride	less than 0.02	0.02

The ideal sample’s GC test results are displayed in Table 10, which shows that the sample complies with EN 14103 (Biodiesel, 2011) and EN 14105 (Biodiesel and Glycerol, 2011) biodiesel requirements.

Based on research done by Kolobeng et al. (2022) who examined the different physical properties of biodiesel during storage, the increase in the moisture content in the biodiesel because of the moisture content of air accelerates biodiesel degradation. The results indicate that the biodiesel produced from waste cooking oil can be stored for 11 days after that its moisture content exceeds threshold of 0.5 mg/g so it must be blended with petroleum diesel before that time to delay the degradation and ensure good quality as indicated by standard ASTM D2709.

### 3.7 Catalyst reusability

**FIGURE 15:**
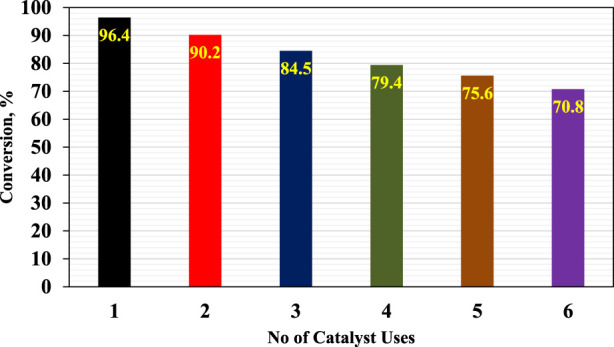
Reusability test of iron fillings solid waste.

The catalyst’s reusability test produced an intriguing discovery. It was discovered that the catalyst may be reused up to six times before it is necessary to replace it. Figure 15, which depicts the trend of catalyst performance through numerous reuse cycles, graphically represents this information. Two major causes can be ascribed to the shift in catalyst reactivity. First, glycerol tends to build on the active centers of the catalyst after repeated application. This glycerol build-up impairs the catalyst’s capacity to carry out the intended reactions. Second, some particle loss may occur in the catalyst during the filtration phase of the process. The catalytic activity of the remaining catalyst material is reduced because of these lost particles. These components work together to explain the observed variations in catalyst reactivity over time. As a result, after a specific number of cycles, it is advised that the catalyst be replaced with a new one. This enables peak performance and the necessary response efficiency.

## 4 Comparison of the current study with the prior studies

**FIGURE 16:**
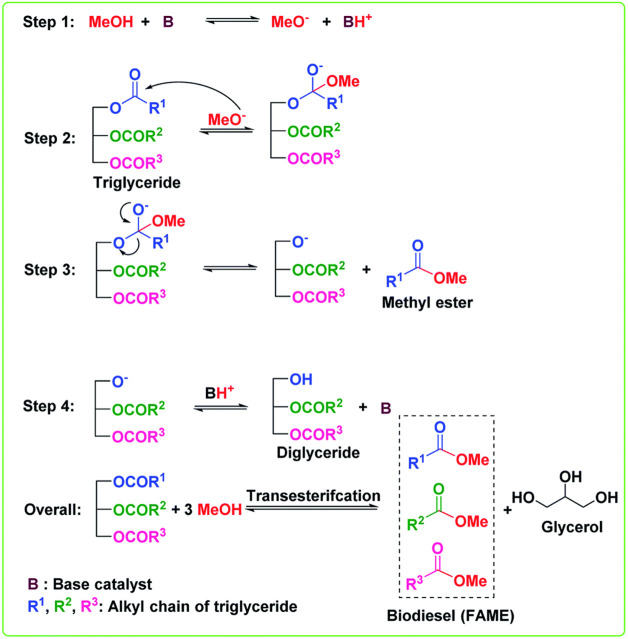
Reaction mechanism.

As seen in Table 11, this study has various distinguishing qualities that set it apart from past studies. The benefits are as follows:

1- A solid waste-derived catalyst has been utilized, which eliminated the need for extra catalyst preparation. This streamlines the procedure and saves time and money.
2- The catalyst utilized in this work is heterogeneous, making separation from the reaction mixture easier. This streamlines the whole method and enables effective catalyst recovery and reuse.
3- The utilization of used cooking oil as a raw source in the creation of biodiesel. The work tackles both waste management problems and taps into a readily available resource by reusing this waste product.
4- The remarkable biodiesel conversion rates observed under minimum reaction conditions distinguish this work. This implies that the procedure uses less energy and is less expensive than other approaches.
5- This study’s emphasis on recycling hazardous solid waste and waste cooking oil for biodiesel generation is one of its most significant contributions. The researchers cut process expenses while simultaneously contributing to environmental conservation initiatives.
6- The iron fillings solid waste contains 96% Fe_2_O_3_ with other minor oxides making it a good biodiesel as iron oxide is proved by previous researchers to be a perfect biodiesel catalyst (Widayat and Nursafitri, 2019; Xie and Wang, 2020a; Dhawane et al., 2016; Basavegowda et al., 2017; Xie and Wang, 2020b; Panchal, 2018; Kumar et al., 2024; Ezzah-Mahmudah et al., 2016; Suzuta et al., 2012; Ibrahim et al., 2022).
7- Iron ferric oxide is considered a heterogeneous basic catalyst that is used to produce biodiesel in which alcohol and edible oil “triglyceride, TG” undergo nucleophilic reactions to form biodiesel “Fatty acid methyl ester, FAME” and glycerol as a byproduct. The mechanism of the transesterification reaction is illustrated in the following figure. Triglyceride is firstly converted to diglyceride then monoglyceride and finally the monoglyceride is converted to glycerol. An ester is formed in each conversion step so on molecule of TG produces three molecules of ester as shown in Figure 16 (Changmai et al., 2020).
8- The study explores the use of waste iron filling as an innovative, low-cost, and substitute high-cost source of ferric compound for the creation than using the resulting iron oxide as a catalyst used in the manufacturing of biodiesel.

**TABLE 11:** Comparison of current research with another research.

Study no.	Catalyst utilized	Preparation method	Reaction parameters				Conversion (%) of biodiesel	Reference
			Methanol to oil molar ratio	Loading wt (%)of catalyst	Reaction temperature (°C)	Reaction time		
1	Fe_2_O_3_/CaO catalyst	Impregnation and calcination methods	15:1	1 wt.	65	3 h	95	Ezzah-Mahmudah et al. (2016)
2	FeOx/SiO_2_ catalyst	Pore-filling method	218:1	15 wt.	220	3 h	99	Suzuta et al. (2012)
3	CaO-Fe_2_O_3_ nanocatalyst	Impregnation method	18:1	3 wt.	65	3 h	98.3	Ibrahim et al. (2022)
4	Iron Fillings Solid Waste (96.15% Fe_2_O_3_)	No need to prepare	30:1	8 wt.	52	1 h	96.4	(Current work)

Overall, this research proposes a viable alternative to biodiesel production by employing a solid waste catalyst, speeding the separation process, utilizing waste cooking oil, attaining high conversion rates, and promoting environmental sustainability. These elements, taken together, constitute a substantial improvement in biodiesel research.

## 5 Conclusion

The research delved into the innovative use of iron filings solid waste as a catalytic agent for biodiesel production, presenting a dual solution to environmental concerns and the quest for renewable energy sources. Through optimizing the biodiesel production process via a transesterification reaction, this study not only proposes a method to mitigate hazardous waste but also enhances the sustainability of energy production. This investigation, focusing on critical reaction parameters—temperature, stirring rate, methanol to oil ratio, catalyst loading, and reaction time—utilized Design Expert software to methodically analyze data, model outcomes, and refine the production process.

Particle size distribution analysis, X-ray diffraction (XRD), scanning electron microscopy (SEM), X-ray fluorescence (XRF), Thermal analysis (TG-DTA), and FTIR analysis were used to characterize the iron filings. These analyses proved that the solid waste contains 96.15% Fe_2_O_3_ with small particle size and large surface area which is a good indication that this solid can be used as a heterogeneous biodiesel catalyst.

The execution of 43 experimental trials, employing a variety of analytical tools such as 3D charts, 2D graphs, and contour figures, allowed for an in-depth examination of each parameter’s influence on biodiesel yield. The process modeling resulted in a reduced quadratic model representing the results with small errors between actual and predicted yield. The results showed that the amount of catalyst added to the reaction mixture and the reaction time have approximately no effect while the reaction temperature, the M:O ratio, and the stirring rate have the greatest impact on both biodiesel and glycerol conversions.

The optimization process resulted in the identification of one hundred potential solutions, balancing environmental impact and economic feasibility, ultimately leading to the selection of the most effective reaction conditions. The study established optimal conditions of a 30:1 methanol to oil molar ratio, 52°C reaction temperature, 8% catalyst loading, 1-h reaction time, and 200 rpm stirring rate, under which a 96.4% biodiesel conversion rate was achieved, fulfilling the set criteria for quality biodiesel. The resulting optimum biodiesel sample was tested by GC and physical properties determination and the results were compared with the American and British standards showing and proofing that the resulting sample is a good biodiesel fuel.

This research not only underscores the potential of repurposing iron filings solid waste for environmental benefit but also highlights the economic advantages of utilizing waste materials as raw inputs for biofuel production. The conclusion of the study, demonstrating the catalyst’s capacity for up to six reuses without significant efficiency loss, emphasizes the method’s practical viability and sustainability. In summary, these findings advocate for further exploration and application of this approach in industrial settings, reinforcing the concept that innovative, green chemistry can play a pivotal role in addressing environmental challenges and advancing renewable energy technologies.

## Data Availability

The original contributions presented in the study are included in the article/supplementary material, further inquiries can be directed to the corresponding authors.
